# Apoptotic stress induces Bax-dependent, caspase-independent redistribution of LINC complex nesprins

**DOI:** 10.1038/s41420-020-00327-6

**Published:** 2020-09-18

**Authors:** Liora Lindenboim, Dan Grozki, Ayelet R. Amsalem-Zafran, Aida Peña-Blanco, Gregg G. Gundersen, Christoph Borner, Didier Hodzic, Ana J. Garcia-Sáez, Howard J. Worman, Reuven Stein

**Affiliations:** 1 Department of Neurobiology, School of Neurobiology, Biochemistry and Biophysics, George S. Wise Faculty of Life Sciences, Tel Aviv University, Ramat Aviv, 69978 Israel; 2 Interfaculty Institute of Biochemistry, University of Tübingen, 72074 Tübingen, Germany; 3 Department of Pathology and Cell Biology, Vagelos College of Physicians and Surgeons, Columbia University, New York, NY 10032 USA; 4 Institute of Molecular Medicine and Cell Research, Albert Ludwigs University of Freiburg, Stefan Meier Strasse 17, D-79104 Freiburg, Germany; 5 Spemann Graduate School of Biology and Medicine (SGBM), Albert Ludwigs University of Freiburg, Albertstrasse 19a, D-79104 Freiburg, Germany; 6 Department of Developmental Biology, Washington University School of Medicine, 660S. Euclid Avenue, St Louis, MO 63110 USA; 7 Institute for Genetics and Cologne Excellence Cluster on Cellular Stress Responses in Aging-Associated Diseases (CECAD), University of Cologne, Joseph-Stelzmann-Strasse 26, 50931 Cologne, Germany; 8 Department of Medicine, Vagelos College of Physicians and Surgeons, Columbia University, New York, NY 10032 USA

**Keywords:** Apoptosis, Nucleoproteins

## Abstract

The canonical function of Bcl-2 family proteins is to regulate mitochondrial membrane integrity. In response to apoptotic signals the multi-domain pro-apoptotic proteins Bax and Bak are activated and perforate the mitochondrial outer membrane by a mechanism which is inhibited by their interaction with pro-survival members of the family. However, other studies have shown that Bax and Bak may have additional, non-canonical functions, which include stress-induced nuclear envelope rupture and discharge of nuclear proteins into the cytosol. We show here that the apoptotic stimuli cisplatin and staurosporine induce a Bax/Bak-dependent degradation and subcellular redistribution of nesprin-1 and nesprin-2 but not nesprin-3, of the linker of nucleoskeleton and cytoskeleton (LINC) complex. The degradation and redistribution were caspase-independent and did not occur in Bax/Bak double knockout (DKO) mouse embryo fibroblasts (MEFs). Re-expression of Bax in Bax/Bak DKO MEFs restored stress-induced redistribution of nesprin-2 by a mechanism which requires Bax membrane localization and integrity of the α helices 5/6, and the Bcl-2 homology 3 (BH3) domain. We found that nesprin-2 interacts with Bax in close proximity to perinuclear mitochondria in mouse and human cells. This interaction requires the mitochondrial targeting and N-terminal region but not the BH3 domain of Bax. Our results identify nesprin-2 as a Bax binding partner and also a new function of Bax in impairing the integrity of the LINC complex.

## Introduction

Apoptosis is a regulated cell death process whose intrinsic pathway is mainly regulated by the action of Bcl-2 family proteins^1^. There are three groups of BCL-2 proteins: pro-survival Bcl-2 proteins, pro-apoptotic Bcl-2 homology 3 (BH3)-only proteins and pro-apoptotic effector proteins (Bax, Bak, and Bok)^2^. Bcl-2 proteins control cell death mainly by regulating Bak and Bak-dependent mitochondrial outer membrane permeabilization (MOMP), which causes release of apoptogenic proteins such as cytochrome *c* from the mitochondrial intermembrane space into the cytosol. This in turn causes caspase activation and cell death^3^. Pro-survival Bcl-2 proteins inhibit MOMP by binding directly to BH3-only proteins or by binding to activated Bax and Bak.

Bcl-2 family proteins also have non-apoptotic functions^4–6^. We previously showed that in response to apoptotic stimuli or forced expression of Bax at the outer membrane of the nuclear envelope (NE), Bax triggers nuclear protein redistribution (NPR)^7,8^. This process involves Bax-regulated disturbances in NE proteins, including lamin A/C, which results in the generation and subsequent rupture of nuclear protein-containing bubbles encapsulated by nuclear pore-depleted NE. We termed this process “stress-induced generation and rupture of nuclear bubbles” (SIGRUNB)^9^. SIGRUNB can be repetitive and ultimately lead to the discharge of nuclear proteins into the cytoplasm. It precedes morphological changes of apoptosis, occurs independently of caspases and cytochrome *c* release and is not inhibited by Bcl-x_L_^9^.

Generation and rupture of nuclear bubbles (GRUNB) also occurs in the absence of exogenous stress. Cultured cells from patients with lamin A/C gene mutations and cells derived from tumors exhibit spontaneous and repeated NE ruptures accompanied by discharge of nuclear proteins into the cytosol^10–12^. GRUNB also occurs in cells expressing the HIV Vpr^13^, in *Drosophila* muscle cells during Wnt signaling^14^, during confined cell migration^15–17^, in response to mechanical compression^18^ and in migrating neurons lacking lamin B1^19^. Notably, spontaneous GRUNB occurring in cultured cancer cells with reduced levels of lamin B1 and in fibroblasts lacking all lamins requires assembly of the linker of nucleoskeleton and cytoskeleton (LINC) complex^20,21^.

The LINC complex mechanically links the nucleus to the cytoskeleton. It is composed of Klarsicht/ANC-1/Syne-1 homology (KASH) domain proteins in the outer nuclear membrane and SUN domain proteins in the inner nuclear membrane^22–24^. The KASH domain of nesprins projects into the perinuclear space, where it interacts with the SUN domain of SUN proteins. KASH domain proteins also extend into the cytoplasm where they interact with cytoskeletal components, thus connecting the cytoskeleton to the SUN proteins in the inner nuclear membrane. SUN proteins in turn interact with A-type lamins, chromatin-binding proteins and other proteins^22^.

In mammals, there are six KASH domain proteins. Two of them, nesprin-1 and nesprin-2, are encoded by genes containing more than 100 exons that lead to multiple isoforms^25,26^. The largest isoforms of nesprin-1 and nesprin-2 are termed nesprin-1-Giant (nesprin-1G) and nesprin-2-Giant (nesprin-2G), respectively. These giant proteins have an N-terminal actin-binding site consisting of paired actin-binding calponin-homology domains, followed by a rod-like structure composed of multiple spectrin-repeats. Binding of nesprin-2G to actin is also facilitated by interactions with FHOD1^27,28^ and fascin^29^. A third smaller protein, nesprin-3, also contains spectrin-repeats. The nesprin-3β isoform binds the cytoskeletal crosslinker protein plectin providing a connection between the NE and intermediate filaments^30^. Given our previous results showing that during apoptotic stress Bax impairs NE integrity, we hypothesized that this effect is associated with impaired integrity of LINC complex.

## Results

Apoptotic stimuli cause Bax/Bak-dependent and caspase-independent redistribution of nesprin-1 and nesprin-2

**Fig. 1:**
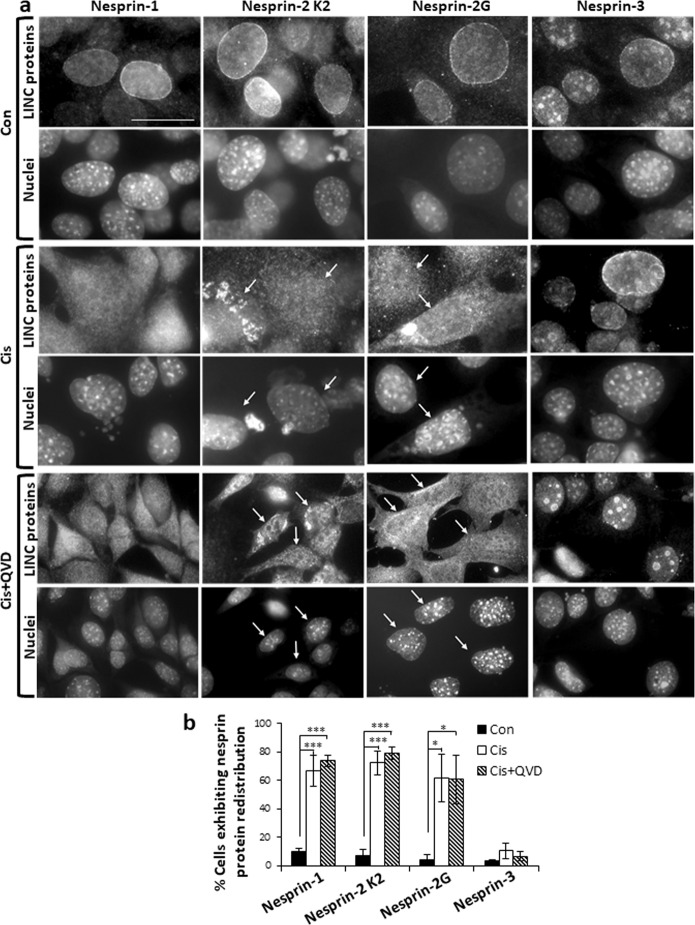
Apoptosis induces caspase-independent redistribution of nesprin-1 and nesprin-2, but not nesprin-3, in WT MEFs. WT MEFs were untreated (Con) or treated for 24 h with 25 µM cisplatin (Cis) without or with Q-VD-OPH (QVD), followed by staining with pan anti-nesprin-1 and nesprin-2 (Nes1 HAA12 and Nesprin-2 K2, respectively) and nesprin-2G or nesprin-3 Ab, and with the Hoechst 33258 dye (for staining the nuclei), and visualized by fluorescence microscopy. **a** Photomicrographs corresponding to each treatment (upper and lower panels) showing the same field visualized separately for detecting Ab and nuclei staining. The results presented are from a representative experiment (one of three independent experiments). Arrows indicate cells exhibiting redistribution and their nuclei. Bar = 25 µm. The intranuclear patches of nesprin-3 are most likely background staining. **b** Quantification of nesprin proteins redistribution. The results presented are expressed as the percentage of cells exhibiting redistribution of each nesprin protein from all the cells counted (at least 200 cells) in each treatment. Values are presented as mean ± SEM (error bars) (*n* = 3). (*p < 0.05, ***p < 0.002; two tailed student’s *t* test).

To assess the effect of apoptotic stimuli on LINC complex integrity, we treated WT MEFs with cisplatin followed by staining with Ab against multiple isoforms of nesprin-1 (Nes1 HAA12^31^) and nesprin-2 (Nes2 K2^31^), against nesprin-2G^32^ and against nesprin-3^33^. In response to cisplatin, both nesprin-1 and nesprin-2 redistributed from the NE to the cytoplasm whereas nesprin-3 did not (Fig. 1a). In WT MEFs, cisplatin treatment significantly increased the percentage of cells exhibiting nesprin-1 or nesprin-2 displacement from the NE but nesprin-3 localization was unaffected (Fig. 1b). We next examined the role of caspases in nesprin redistribution. Treatment of WT MEFs with the pan caspase inhibitor Q-VD-OPH did not affect the cisplatin-induced redistribution of nesprin-1 or nesprin-2 (Fig. 1a, b). For confirmation, we used *caspase-9*^*−/−*^ MEFs, which lack apoptosome-mediated caspase activation. The lack of capsase-9 did not prevent cisplatin-induced nesprin-1 and nesprin-2 redistribution (Fig. S1a and b). Similar results were obtained when *caspase-9*^*−/−*^ MEFs were treated with staurosporine, a different apoptotic stimulus (Fig. S1a and b).

**Fig. 2:**
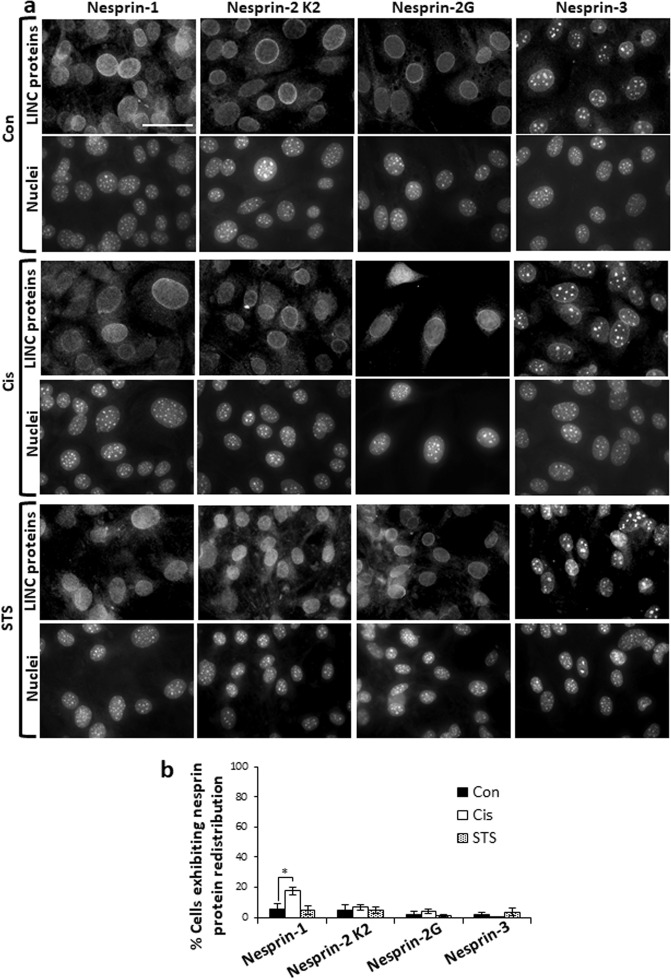
Apoptotic stimuli do not cause nesprin-1 and nesprin-2 redistribution in Bax/Bak DKO MEFs. Bax/Bak DKO MEFs were untreated (Con) or treated for 24 h with 25 µM cisplatin (Cis) or 17 h with 100 nM staurosporine (STS). Cells were then stained for nesprin proteins as described in Fig. 1. **a** Representative photomicrographs of the staining of the different nesprin proteins. Bar = 50 µm. **b** Quantification of each nesprin protein redistribution expressed as described in Fig. 1. Values are presented as mean ± SEM (error bars) (*n* = 3) (*p < 0.05, two tailed student’s *t* test).

We next studied the role of Bax/Bak in nesprin redistribution by treating Bax/Bak double knockout (DKO) MEFs with cisplatin or staurosporine. Only nesprin-1 was minimally redistributed following cisplatin treatment (Fig. 2a, b). These results demonstrated that two different apoptotic stimuli cause the redistribution of nesprin-1 and nesprin-2 from the NE to the cytoplasm in a Bax/Bak-dependent, but caspase-independent manner.

**Fig. 3:**
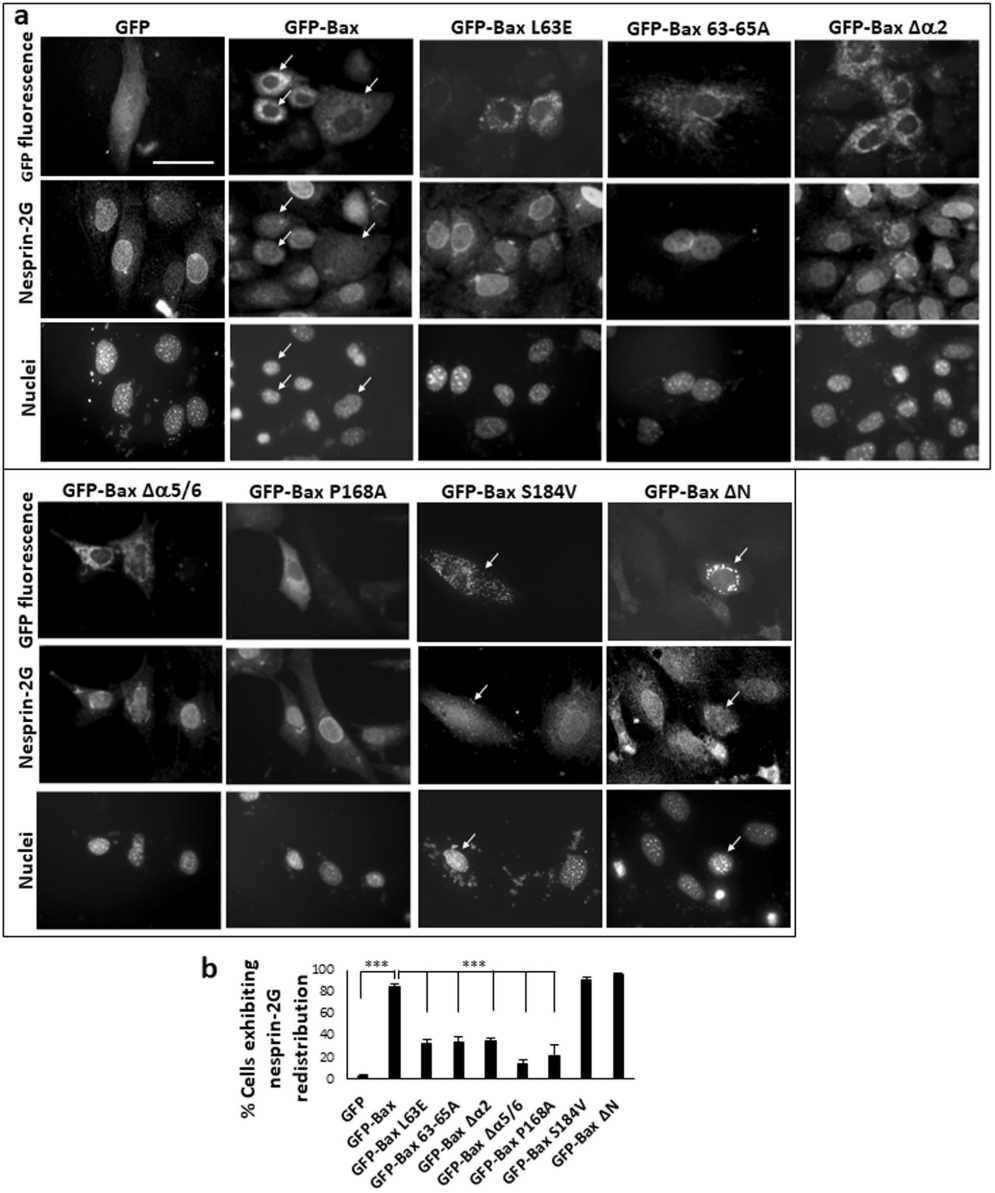
Identification of Bax domains needed for nesprin-2G redistribution. **a** Bax/Bak DKO MEFs were transfected with GFP, GFP-Bax, or the indicated GFP-Bax mutants in the presence of Q-VD-OPH. 24 h later the cells were stained with anti-nesprin-2G Ab and Hoechst dye, and visualized by fluorescence microscopy. The photomicrographs of each treatment represent the same field visualized separately for the detection of GFP fluorescence, nesprin-2G staining and Hoechst-stained nuclei. The results presented are from a representative experiment (out of three independent experiments). Bar = 50 µm. Arrows indicate representative cells and their nuclei exhibiting nesprin-2G redistribution. **b** Quantification of the percentage of GFP, GFP-Bax, or GFP-Bax mutants-expressing cells exhibiting nesprin-2G redistribution. The results presented are expressed as percentage of cells exhibiting both nesprin-2G redistribution and GFP, GFP-Bax, or GFP-Bax mutants’ expression, from the total population of GFP-expressing cells (at least 100 cells). Values are presented as mean ± SEM (error bars) (*n* = 3). (***p < 0.002, two tailed student’s *t* test between GFP and GFP-Bax). A one-way ANOVA followed by *Dunnett* post hoc test revealed significance between GFP-Bax and the indicated Bax mutants (***p < 0.002).

To further substantiate the role of Bax in the NE to cytoplasmic redistribution of nesprins, we expressed Bax in Bax/Bak DKO MEFs using a doxycycline-inducible cell line (C16 DKO MEFs). After doxycycline treatment, the C16 DKO MEFs were treated with cisplatin in the presence of Q-VD-OPH. Expression of Bax promoted nesprin-1 and nesprin-2, but not nesprin-3, redistribution in a similar manner as in cisplatin-treated WT MEFs (Fig. S2). These results suggest that nesprin-1 and nesprin-2 nuclear/NE-cytoplasmic redistribution in response to apoptotic stimuli is mediated via a Bax-dependent signaling pathway. We next wanted to identify the Bax domains required for nesprin redistribution. To do so, we transfected Bax/Bak DKO MEFs with expression vectors encoding WT GFP-Bax or GFP-Bax variants, in which various functional domains/helices were mutated or deleted^8^. The transfection stress led to a modest redistribution of nesprin-2G in the GFP transfected cells; however, expression of WT Bax substantially enhanced nesprin-2G redistribution, as indicated by appearance of nesprin-2G in the cytoplasm and its disappearance from the NE (Fig. 3). This Bax-induced nesprin-2G redistribution effect was unaffected by deleting the first 20 amino acids [∆N(1–20) Bax] or by targeting Bax (S184V) to mitochondria^34^. However, substitutions of critical amino acids in the BH3 domain (L63E) or in the region essential for homo-oligomerization (63–65 A), or deleting the entire BH3 domain [depleting helix 2 (∆α2)] or helices 5 and 6 in the core and latch domains (∆α5/6), or targeting Bax to the cytosol (P168A^35^) reduced the ability of Bax to promote nesprin-2G redistribution. These results suggest that nesprin-2G redistribution depends on the membrane localization of Bax, α helices 5/6 and the BH3 domain, all also known to be required for MOMP.

**Fig. 4:**
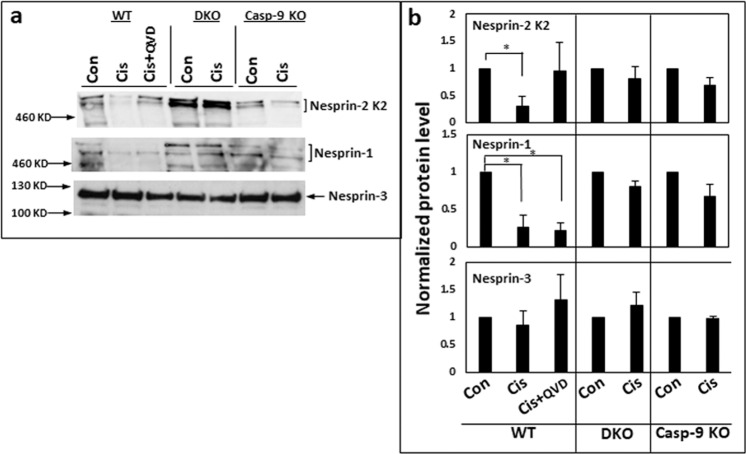
Immunoblot analysis of the expression of the LINC complex proteins: nesprin-1, nesprin-2, and nesprin-3, in cisplatin-treated WT, Bax/Bak DKO and *caspase-9*^*−/−*^ MEFs. Protein samples were prepared from WT MEFs untreated (Con) or treated for 24 h with 25 µM cisplatin (Cis) or with cisplatin with the presence of Q-VD-OPH (Cis + QVD) or from *caspase-9*^*−/−*^ and Bax/Bak DKO MEFs untreated (Con) or treated for 24 h with 25 µM cisplatin, were separated on gradient SDS-PAGE, blotted and probed for nesprin-2, nesprin-1 and nesprin-3 expression. **a** Images of a representative blot for each protein in the different cell types. **b** Quantification of the levels of the different proteins from all experiments. Nesprin levels were normalized (as described in “Materials and Methods” section) to 40 kDa and 80 kDa bands in Ponceau staining (Fig. S3) as internal loading controls. The results are presented as the normalized protein values relative to their corresponding controls (value 1) and are expressed as mean ± SEM (error bars) (*n* = 4). (*p < 0.05, two tailed student’s one sample *t*-test).

We examined the effect of an apoptotic stress on the degradation of nesprin-1, nesprin-2, and nesprin-3. In protein extracts of WT MEFs analyzed by immunoblotting, cisplatin treatment led to diminishment of the high molecular mass protein bands corresponding to nesprin-2 and nesprin-1 but not nesprin-3 (Fig. 4 and Fig. S3). However, while the diminishment of nesprin-2 was inhibited by Q-VD-OPH, that of nesprin-1 was not. In contrast, this diminishment was not observed in cisplatin-treated Bax/Bak DKO or *caspase-9*^*−/−*^ MEFs. This finding indicates that in addition to inducing nesprin redistribution, apoptotic stimuli may impair the LINC complex by inducing Bax/Bak- and caspase-9-dependent degradation of nesprin-1 and nesprin-2, but not of nesprin-3.

To unravel a potential role for nesprin-2 redistribution in the apoptotic process, we used confocal fluorescence microscopy to assess its localization after treatment of *caspase-9*^*−/−*^ MEFs with cisplatin. These MEFs enable assessing localization in stressed, but viable cells. Examination of cells co-labeled with anti-nesprin-2 Ab, MitoTracker Red and Ab against active Bax (6A7) showed that Bax and the redistributed nesprin- co-localized in close proximity to perinuclear mitochondria (Fig. S4). Hence, redistributed nesprin-2 and Bax may interact in close proximity to mitochondria.

### Bax interacts with nesprin-2 and the interaction increases in response to apoptotic stimuli

**Fig. 5:**
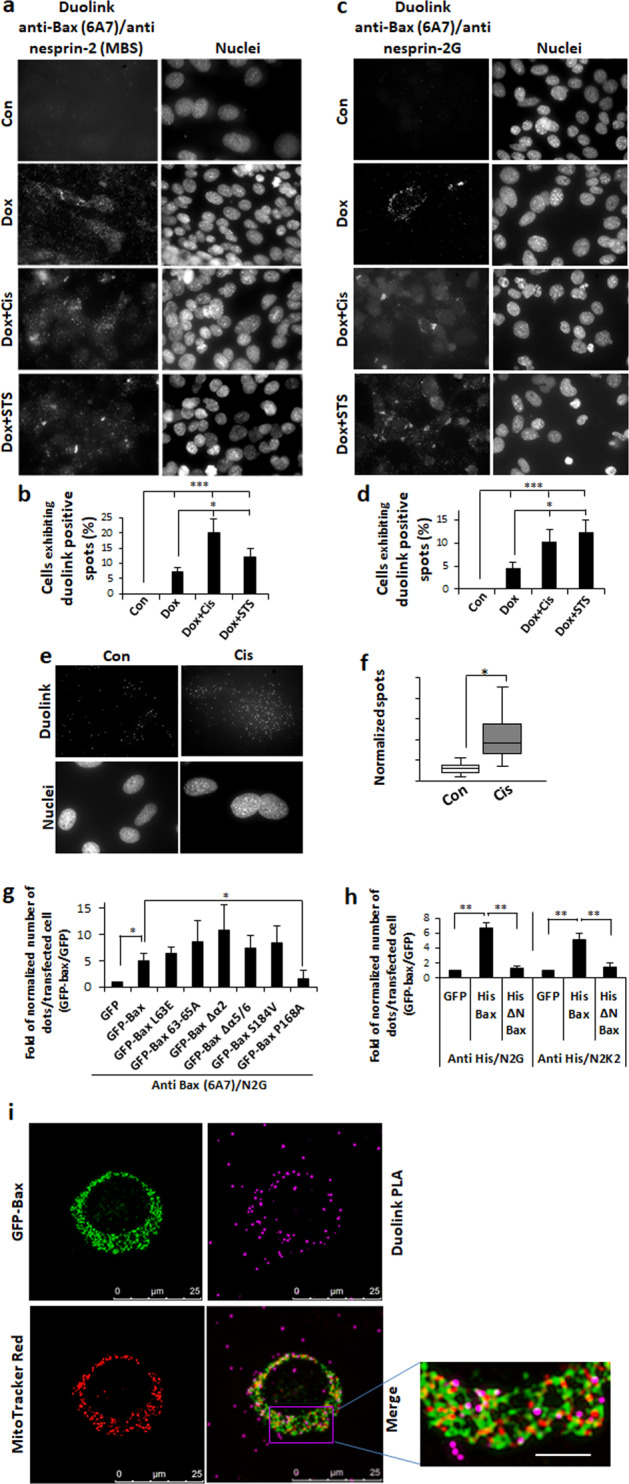
Bax can interact with nesprin-2; Duolink PLA approach. **a**–**d** Inducible Bax interacts with nesprin-2. Bax/Bak C16 DKO MEFs were untreated (Con) or treated for 24 h with doxycycline to induce Bax expression (Dox), or treated for 24 h with doxycycline followed by addition of 25 µM cisplatin (Dox + Cis) or 100 nM staurosporine (Dox + STS) in the presence of Q-VD-OPH for additional 24 h and 17 h, respectively. Duolink-PLA was preformed using anti-Bax 6A7 Ab together with anti-nesprin-2 MBS or anti-nesprin-2G Ab. Cells were visualized using fluorescence microscopy. **a** Representative photomicrographs of cells showing Duolink signals using anti-nesprin-2 MBS. The images represent the same field visualized separately for detecting Duolink signal (left panel) and nuclei (DAPI) (right panel) staining. Arrows indicate cells which exhibit Duolink signal that accumulates in aggregates. Bar = 50 µm. **b** Quantification of the percentage of cells exhibiting Duolink signal using anti-nesprin-2 MBS from total number of cells. Cells were detected by their nuclei and at least 200 cells were analyzed in each treatment. The values are represented as mean ± SEM (error bars) (*n* = 3). (*p < 0.05, ***p < 0.002; one tailed student’s *t* test compared to con*t*rol and **p < 0.002 compared to doxycycline treatment). (**c**) Representative photomicrographs of cells showing Duolink signals using anti-nesprin-2G Ab. The images are shown as described in **a**. **d** Quantification of the percentage of cells exhibiting Duolink signal using anti-nesprin-2G Ab from total number of cells. The results are presented as described in **b**. **e**, **f** Interaction between endogenous Bax and nesprin-2. *Caspase-9*^−/−^ MEFs were untreated (Con) or treated with 25 µM cisplatin (Cis). Duolink signal was detected using anti-active Bax Ab (6A7) and pan anti-nesprin-2 (MBS375177) Ab. **e** Duolink signal in each treatment was visualized using fluorescence microscopy. The photomicrographs shown for each treatment are from the same field visualized separately for detecting Duolink signal and nuclei. Bar = 50 µm. **f** Quantification of the Duolink signal. The presented results are expressed as the number of Duolink dots (Duolink signal) per captured field after normalization to the number of nuclei in the field (12-19 fields per experiment; *n* = 2). Two-way ANOVA after log transformation for normal distribution show significant difference between the two treatments (*p* < 0.0001). **g**, **h** Identification of Bax domains needed for Bax/nesprin-2 interaction. Bax/Bak DKO MEFs were transiently transfected with control GFP (**g**), or His-tagged (**h**) expression vectors or with expression vectors for WT Bax or Bax mutants, in the presence of Q-VD-OPH. Duolink PLA assay was preformed using the anti-Bax (6A7) Ab [for GFP-Bax (**g**)] or anti-His Ab [for His-Bax (**h**)] and anti-nesprin-2G Ab (N2G) or pan anti-nesprin-2 K2 (N2K2) Ab. Cells exhibiting Duolink signal were visualized using fluorescence microscopy. The number of Duolink dots displayed by the transfected cells (at least 20 cells for each treatment) were normalized to cell size, expressed relative to GFP-transfected cells and presented as mean ± SEM (error bars) (*n* = 3). (*p < 0.05, **p < 0.02, two tailed student’s *t* test comparing GFP-Bax or His-Bax to GFP, or His-Bax *t*o His ΔN-Bax and one sample one tailed student’s *t* test comparing GFP-Bax to GFP-Bax P168A). **i** Localization of Bax and nesprin-2 interaction site. Bax/Bak DKO MEFs were transiently transfected with GFP-Bax, stained with MitoTracker Red, fixed and assessed for interaction between GFP-Bax and endogenous nesprin-2G by Duolink-PLA using anti-Bax (6A7) and anti-nesprin-2G Ab. Photomicrographs were captured by confocal microscopy. The results shown are from a representative cell (out of 29 cells from 6 independent experiments). Bar = 25 µm. The same field was visualized separately for detection of GFP-Bax (green), Duolink signal (Magenta), and mitochondria (red) fluorescence. Higher magnification of a representative area (denoted by a box in the merge image) in the merge image illustrates close proximity of Duolink dots with GFP-Bax and mitochondria. Bar = 5 µm.

We first assessed a potential Bax/nepsrin-2 interaction in using Duolink PLA in C16 DKO MEFs. Bax expression was induced in these cells and then they were treated with staurosporine or cisplatin in the presence of Q-VD-OPH. The interaction was assessed using anti-active Bax Ab together with either anti-nesprin-2 Ab that detect multiple nesprin-2 isoforms or with Ab which only detect nesprin-2G^32^. Following induction of Bax expression, interactions with nesprin-2 appeared in some cells and in greater numbers in cisplatin- and staurosporine-treated cells. The Duolink signal in many cells accumulated in aggregates. Similar results were obtained when the interaction was assessed using either the pan nesprin-2 Ab (Fig. 5a, b) or the nesprin-2G-specific Ab (Fig. 5c, d). To determine if endogenous nesprin-2 interacts with endogenous Bax, we used *caspase-9*^−/−^ MEFs. Untreated cells contained only a low amount of Duolink signal; however, following cisplatin treatment, the number of Duolink dots increased (Fig. 5e, f). We next examined which Bax regions are required for interaction with nesprin-2. Bax/Bak DKO MEFs were transfected with expression vectors encoding GFP or His-tagged WT Bax or Bax mutants in the presence of Q-VD-OPH. The GFP tagged-Bax mutants L63E, 63–65 A, ∆α2, ∆α5/6, and S184V interacted with nesprin-2G to a similar extent as WT Bax; however, the interaction of P168A was significantly reduced (Fig. 5g). The interaction of Bax lacking its N-terminus [∆N(1-20)] with nesprin-2 was also significantly reduced as assessed using two different anti-nesprin Abs (Fig. 5h). These results indicated that Bax domains associated with MOMP induction are not essential for its binding to nesprin-2G. In contrast, cytosolic Bax (P168A) could not bind to nesprin-2G and the N-terminal region of Bax was needed for the interaction. To characterize where within the cell Bax and nesprin-2 interact, we transfected Bax/Bak DKO MEFs with a GFP-Bax expression plasmid. The subcellular localizations of GFP-Bax, Duolink dots (using anti-Bax 6A7 and anti-nesprin-2G Abs) and mitochondria were then assessed by confocal microscopy. Many Duolink dots had a perinuclear localization in close proximity to mitochondria and GFP-Bax (Fig. 5i).

We also used Duolink-PLA to determine if Bax interacts with nesprin-2 in human U2OS and HCT116 cells. First, we confirmed that these cells express endogenous nesprin-2G by immunostaining and immunoblotting (Fig. S5). Bax/Bak DKO U2OS^36^ and HCT116^37^ cells were then transiently transfected with GFP or GFP-Bax expression vectors and the interaction between transfected Bax and nesprin-2G examined. The amount of Duolink dots was significantly higher in GFP-Bax-expressing Bax/Bak DKO U2OS and HCT116 cells than in control GFP-expressing cells (Fig. S6).

**Fig. 6:**
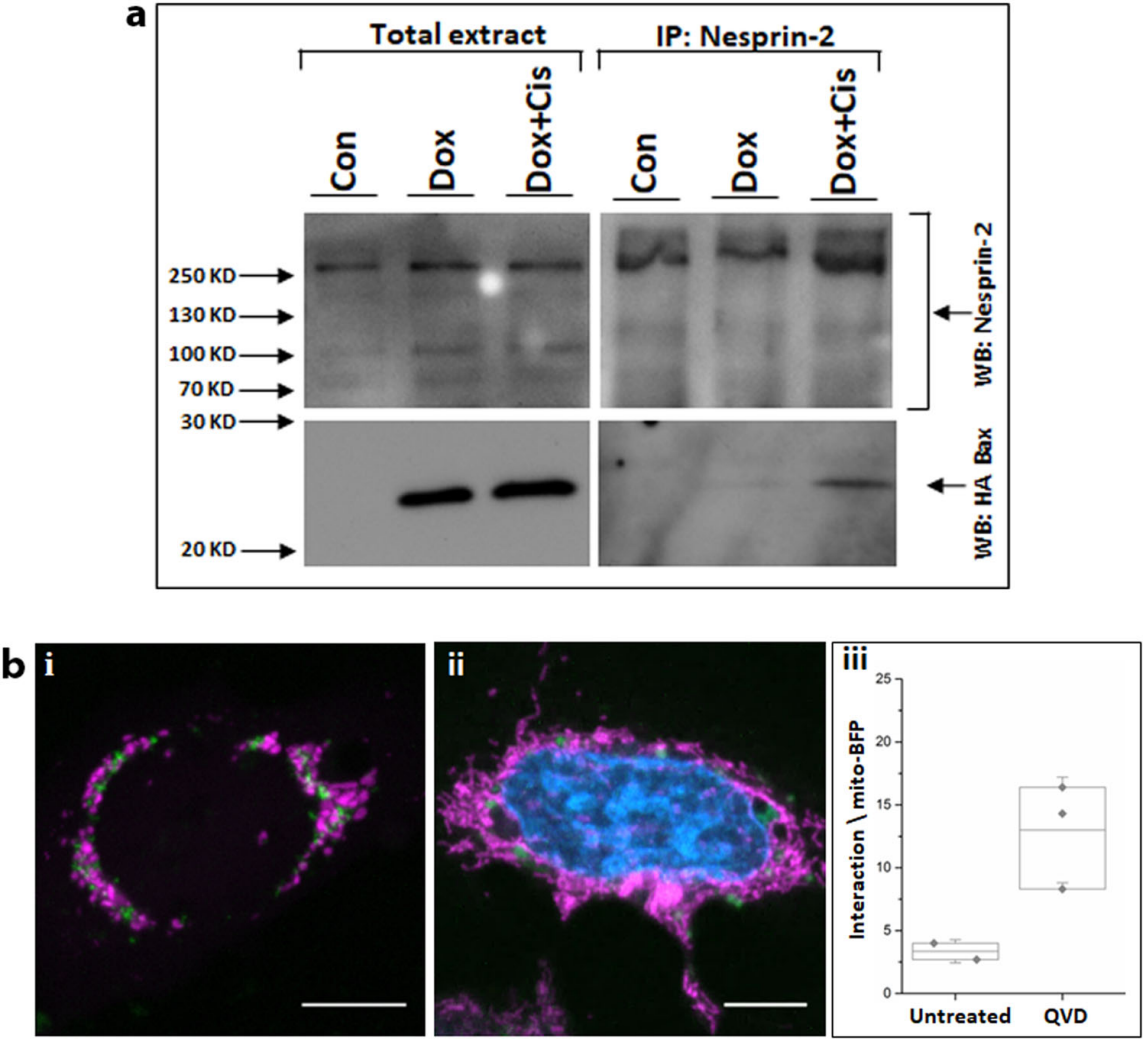
Bax interacts with nesprin-2. **a** Co-IP approach. C16 DKO MEFs were untreated (Con) or treated for 24 h with doxycycline (Dox), or treated for 24 h with doxycycline followed by addition of 25 µM cisplatin in the presence of Q-VD-OPH for additional 24 h (Dox + Cis). IP was performed on the indicted cell lysates using pan anti-nesprin-2 Ab (Santa Cruz). Co-IP of HA-Bax was detected by probing the blots with anti-HA Ab. Nesprin-2 Ab (Santa Cruz) were used to detect the immunoprecipitated nesprin-2. Total cell extracts were immunoblotted with anti-HA or anti-nesprin-2 Ab. Similar results were obtained in an additional independent experiment; (**b**) ddFPX approach. Bax/Bak DKO U2OS cells were transiently transfected with RA-Bax, GB-mini-nesprin-2G and mito-BFP vectors and untreated or treated with 20 µM Q-VD-OPH. (i) Mitochondria localization detected by mito-BFP fluorescence signal (pink). (ii) Mitochondrial (pink) and nuclear (DAPI, blue) fluorescence signal. Dimerization (interaction) of RA-Bax and GB-mini-nesprin-2G is represented by green dots in each panel. Two representative cells are shown. Bar = 10 µm. (iii) Quantification of the interaction signal. The results are expressed as percentage of interacting cells normalized to transfected cells detected by mito-BFP signal. Box plots represent the interquartile (box), mean (line), and standard deviation (whiskers) of three independent experiments (dots); at least 50 cells were counted per repetition.

We next tested the ability of Bax to interact with nesprin-2 by performing co-IP in C16 DKO MEFs. Bax expression was induced with doxycycline and the cells were then treated with cisplatin in the presence of Q-VD-OPH. Bax co-immunoprecipitated with nesprin-2 and this amount was increased by treatment with cisplatin (Fig. 6a). Since nesprin-2 and Bax were expressed at similar levels in untreated and cisplatin-treated cells, the co-IP results indicate that the Bax/nesprin-2 interaction is enhanced by apoptotic stimuli.

We used ddFPX^38^ as a third approach to confirm an interaction between Bax and nesprin-2. In ddFPX, the proteins of interest are tagged with copy A (RA) and copy B (GB), which only fluoresce when they are part of a complex^38^. This approach requires the expression of nesprin-2G cDNA fused to GB. Nesprin-2G contains 6874 amino acids, making cloning of its full-length cDNA difficult. We therefore used mini-nesprin-2G^39^, which lacks most of the spectrin repeats, but binds to actin and SUN proteins and rescues actin-dependent nuclear movement defects in fibroblasts depleted of nesprin-2G^32^. First, we confirmed that mini-nesprin-2G binds Bax using Duolink PLA. Bax/Bak DKO MEFs were transfected with a plasmids that expresses GFP-mini-nesprin-2G or co-transfected with GFP-mini-nesprin-2G or GFP and FLAG-Bax expression plasmids in the presence of Q-VD-OPH. The interaction was assessed using anti-nesprin-2 K2 and anti-Bax 6A7 Ab. The amount of Duolink dots in cells expressing GFP-mini-nesprin-2G was significantly higher than in control cells expressing GFP (Fig. S7). This demonstrates that mini-nesprin-2G contains the region that interacts with Bax and can be used to assess the interaction using ddFPX. We therefore transfected U2OS Bax/Bak DKO cells, untreated or treated with Q-VD-OPH, with plasmids that express RA-Bax, GB-mini-nesprin-2G and the mitochondrial marker mitoBFP. Analysis of the fluorescence signals showed an interaction between RA-Bax and GB-mini-nesprin-2G. (Fig. 6b, i and ii, green dots). The interaction signal was higher in the Q-VD-OPH treated cells than untreated cells (Fig. 6b, iii). These results further demonstrated a Bax/nesprin-2G interaction that is enhanced when the death effect of Bax and transfection stress are attenuated by Q-VD-OPH. Consistent with the results obtained in MEFs using the Duolink approach, ddFPX confirmed that the Bax/nesprin-2 interaction was associated with perinuclear mitochondria.

### Dislodgment of nesprin-2 from the NE is not sufficient to promote interaction between nesprin-2 and active Bax

**Fig. 7:**
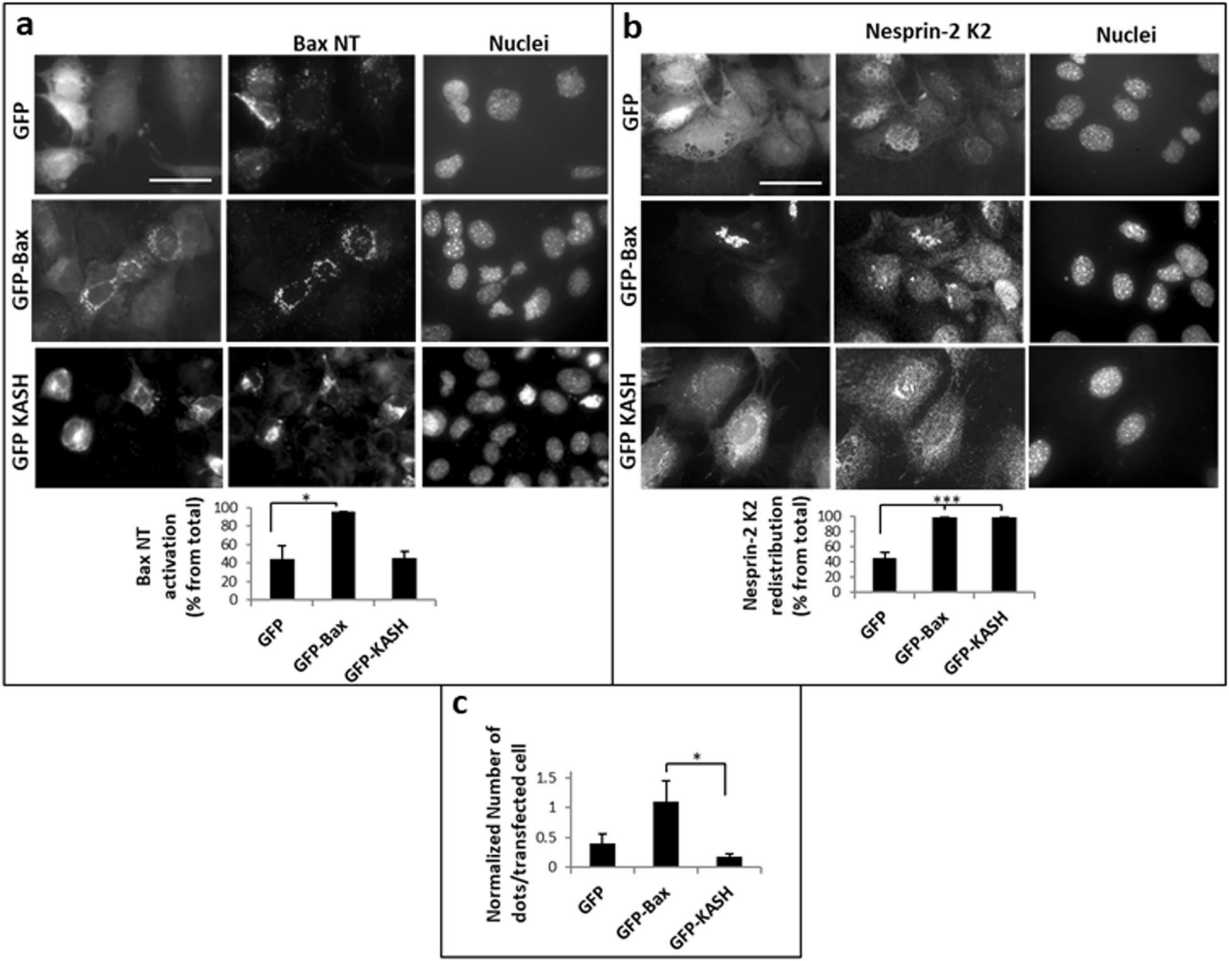
GFP-KASH-induced nesprin-2 redistribution is insufficient to promote interaction between nesprin-2 and Bax. *Caspase-9*^−/−^ MEFs were transfected with GFP, GFP-Bax or GFP-KASH expression vectors. 24 h later, cells were assessed for Bax-NT exposure using anti-Bax (6A7) Ab (**a**), nesprin-2 localization using anti-nesprin-2 K2 (**b**) and for interaction between nesprin-2G and Bax using the Duolink-PLA approach (**c**). The photomicrographs in **a** and **b** of each treatment represent the same field visualized separately for detection of GFP fluorescence, Bax-NT or nesprin-2 K2 staining and Hoechst-stained nuclei. Presented results are from a representative experiment (*n* = 4). Bar = 50 µm. Quantification of the percentage of GFP, GFP-Bax, or GFP-KASH expressing cells, exhibiting Bax-NT exposure or nesprin-2 K2 redistribution from the total population of cells exhibiting GFP fluorescence (total) (at least 50 cells) is shown under the corresponding images. Values are presented as mean ± SEM (error bars) (*n* = 4). (*p < 0.05, ***p < 0.002; two tailed student’s *t* test). (**c**) Quantifica*t*ion of the interaction between nesprin-2G and Bax. Duolink PLA assay was preformed using the Bax (6A7) Ab and anti-nesprin-2G Ab. The results are expressed as the number of dots in transfected cell (at least 20 cells for each treatment) normalized to cell size. Values are presented as mean ± SEM (error bars) (*n* = 4). (*p < 0.05, two tailed student’s *t* test between GFP-Bax and GFP-KASH treatments).

Nesprin-2 redistribution during apoptosis may make it more accessible to interact with Bax. However, the observation that Bax Δα5/6 and Bax ∆BH3 do not promote nesprin-2 redistribution but still interact with it argues against this. We nevertheless assessed Bax/nesprin-2 interaction by inducing nesprin-2 redistribution in another way, i.e., disrupting its interaction with SUN proteins by expressing GFP-KASH^40^. *Caspase-9*^*−/−*^ MEFs were transfected with a GFP-KASH, GFP or GFP-Bax expression vectors. After 24 h, Bax activation (exposure of its N-terminus), nesprin-2 localization and interaction between endogenous nesprin-2 and Bax were assessed in the transfected cells. Duolink interaction assay was performed using anti-Bax 6A7 and anti-nesprin-2G Ab. 6A7 Ab was used to detect endogenous Bax since the transfection stress induces Bax activation, which can be detected by the 6A7 Ab (Fig. 7a). As shown in other cell types^32,40,41^, expression of GFP-KASH induced nesprin-2 redistribution in *caspase-9*^*−/−*^ MEFs (Fig. 7b). However, this redistribution was not accompanied by an increase in the amount Duolink signal compared to that in control GFP-expressing cells and it was significantly lower than the signal in GFP-Bax-expressing cells (Fig. 7c). Thus, nesprin-2 redistribution per se is not sufficient to promote its interaction with active Bax.

## Discussion

**Fig. 8:**
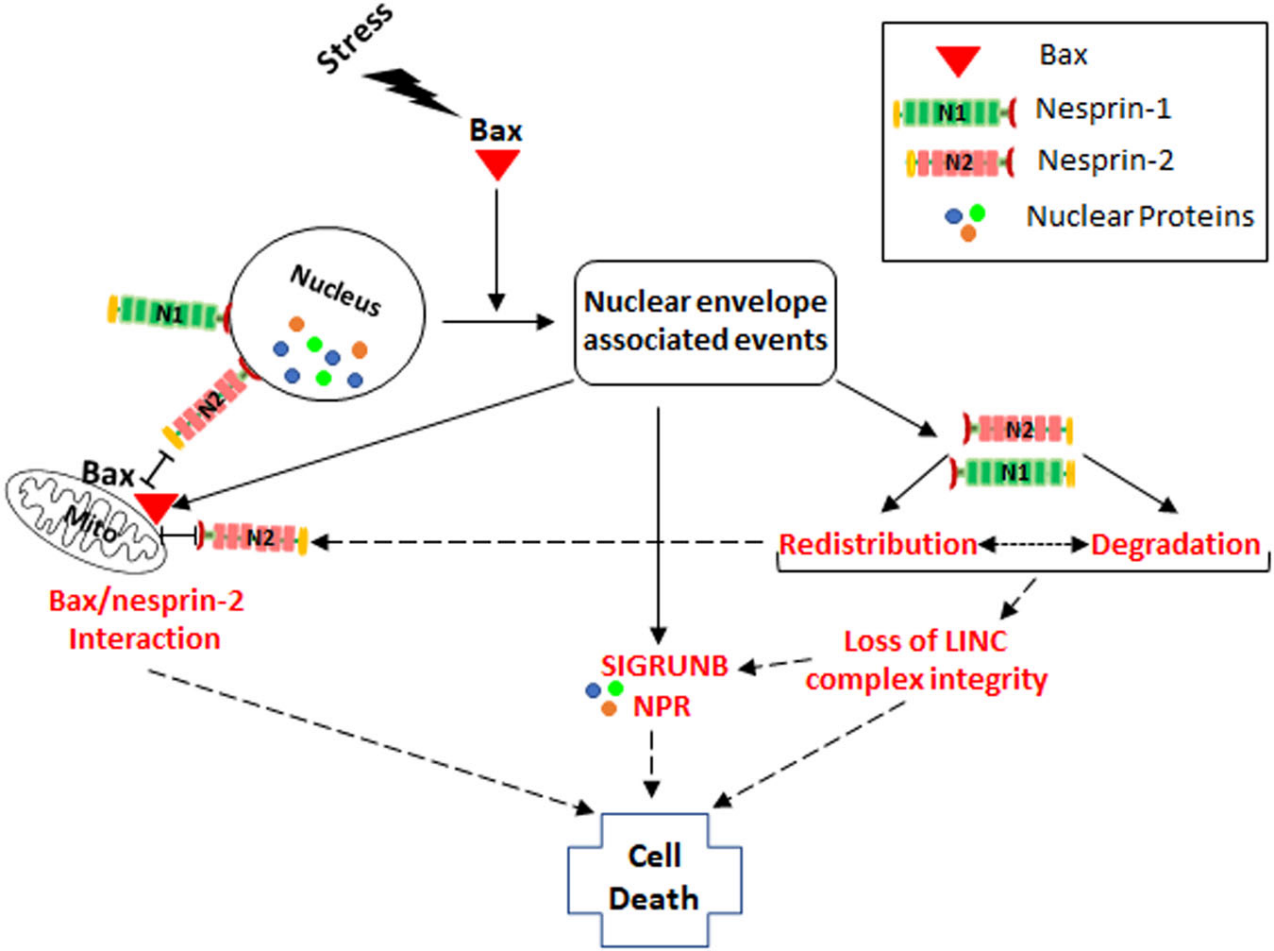
Scheme of stress-induced, Bax-dependent, NE-associated events and their potential interplay in mediating SIGRUNB/NPR and cell death. Apoptotic stresses promote Bax-induced SIGRUNB/NPR. The mechanism whereby Bax promotes SIGUNB/NPR may involve a direct rupture of the NE or nesprin-1 and 2 redistribution, which may lead to their degradation. Alternatively, degradation of nesprin-1 but not nesprin-2 may lead to nesprin-1 redistribution. This results in loss of integrity of LINC complexes containing nesprin-1/2. The stress also promotes an interaction between Bax and nesprin-2, which require Bax-NT exposure and occurs mainly in close proximity to perinuclear mitochondria. The nesprin-2 that interacts with Bax may be within the NE or redistributed. All of these Bax-mediated effects may contribute to cell death either via SIGRUNB/NPR^42^, by abolishing the ability of LINC complexes to connect the nucleus to the certain cytoskeleton elements and by affecting Bax-induced MOMP. Dashed arrows represent the potential pathways.

The LINC complex bridges the nucleus interior to the cytoskeleton and plays important roles in many cellular functions. However, whether the LINC complex has a particular function in stressed cells and what happens to it during apoptotic stimulation has not been studied. We show that apoptotic stimuli such as cisplatin and staurosporine cause redistribution of nesprin-1 and nesprin-2, in a Bax-dependent, caspase-independent manner. In addition, nesprin-1 and nesprin-2 appear to be degraded in a caspase-9-dependent manner. The redistribution and degradation, which do not result from the general destruction of the NE as they are not observed for nesprin-3, lead to loss of integrity of nesprin-1/2-containing LINC complexes. Nesprin-2 also binds to Bax in its active, N-terminus-exposed conformation and this interaction increases in response to apoptotic signals and occurs in close proximity to the perinuclear mitochondria. These Bax-dependent, NE-associated events (described in more detail below) and their potential interplay may contribute to SIGRUNB/NPR and cell death (Fig. 8).

### Apoptotic stimuli impair LINC complex integrity

The apoptosis-induced degradation of nesprin-1 and nespin-2 suggests that this event may lead to their dislodgement and redistribution from the NE. At least for nesprin-2, this does not seem to be the case since its degradation/cleavage, but not redistribution, were prevented by caspase inhibition. With nesprin-1, neither degradation/cleavage nor redistribution were caspase-dependent indicating that both events may be due to another proteolytic event during apoptosis. Strangely, caspase-9 is involved in the nesprin-1 cleavage, but not its redistribution, as it does not occur in *caspase-9*^*−/−*^ MEFs. Why caspase inhibition cannot also block this event is currently unknown. Caspase-9 may have an additional effect on nesprin-1 cleavage, independent of apoptosome-mediated caspase-3 activation.

We showed previously that during apoptosis, proteins are discharged from the nucleus to the cytosol via Bax-dependent NPR/SIGRUNB^7–9^. The Bax pathway which causes nesprin-2 redistribution is similar to that which causes NPR/SIGRUNB. In both cases, it is caspase-independent and requires Bax’s membrane localization, α helices 5/6, and the BH3 domain, implying that NPR/SIGRUNB occurs due to the impairment or modulation of the LINC complex. The effect of apoptosis on the LINC complex is in line with previous findings showing that the NE is an important target of the apoptotic machinery (for review see ref. ^42^).

### Interaction between Bax and nesprin-2

Our results show that Bax can interact with nesprin-2. This interaction increases in response to apoptotic stimuli and engages active Bax. This finding implies that apoptotic stress first promotes the Bax conformational change, which exposes it N-terminus. This notion is supported by the finding that deletion of the first 20 amino acid of Bax inhibits binding to nesprin-2. This is not due to inhibition of Bax translocation to the mitochondria, because deletion of the N-terminus results in auto-insertion into the mitochondrial membrane^43^. Thus, the Bax N-terminus is likely the region that binds nesprin-2.

Bax mutations in regions which are needed for Bax dimerization and oligomerization and subsequent MOMP do not affect Bax/nesprin-2 interaction, indicating that these region are not needed for the interaction. However, preventing translocation of Bax to the mitochondria (P168A Bax) substantially reduces the interaction. This suggests that Bax needs to be targeted to mitochondrial membranes to bind nesprin-2. However, we cannot exclude the possibility that the Bax P168A mutation prevents the interaction by other mean(s), such as altering the conformation of the α8-α9 loop^35^ or by stabilizing inactive dimers in the cytosol^44^ that cannot bind nesprin-2.

### Functional role of the Bax/nesprin-2 interaction in stressed cells

Bax and nesprin-2 interact in close proximity with perinuclear mitochondria, indicating that the interaction may play a role in the apoptotic effect on mitochondria. However, the interaction does not seem to be essential for prompting MOMP because Bax lacking its N-terminus, which does not bind nesprin-2, promotes MOMP (data not shown) and cell death^8,43^. Alternatively, the Bax/nesprin-2 interaction may play a role in non-classical Bax functions in stressed cells. It may modulate the functionality of the LINC complex, which in turn may lead to the observed Bax-dependent effects on the redistribution and degradation of nesprin-2, as well as on SIGRUNB/NPR^8,9^. However, the finding that Bax lacking it N-terminus can still cause nesprin-2 redistribution and NPR does not support such a role, although we cannot exclude the possibility that these effects are mediated by multiple independent effects of Bax.

The nesprin-2 gene encodes multiple isoforms. These isoforms vary in size and subcellular localization. Our results show that Bax can interact with nesprin-2G. However, we cannot exclude the possibility that Bax can interact with other nesprins isoforms and that such interaction(s) might have other functions and require other regions of the protein. Currently, the region in nesprin-2G that interacts with Bax is not known. However, since mini-nesprin-2G binds Bax, that region(s) must be within the 1-485 N-terminal amino acids and/or the 6525–6874 C-terminal amino acids of nesprin 2G.

The Bax/nesprin-2G interaction is predominantly in close proximity to the perinuclear mitochondria. This raise the question of how nesprin-2G gets to the mitochondria. One explanation could be that the stress-induced redistributed nesprin-2G accumulates in close proximity to the perinuclear mitochondria, where it can interact with Bax following Bax translocation to the mitochondria. However, nesprin-2 dislodgment from the NE per se is not sufficient for interaction between endogenous Bax and nesprin-2. Disrupting nesprin-2’s interaction with SUN proteins using GFP-KASH, which redistributes the protein to the endoplasmic reticulum membrane, did not promote its interaction with Bax. This indicates that additional stress signal(s) are needed to promote the interaction. These stress signals may act directly on nesprin-2 or on the cytoskeleton, which in turn may bring nesprin-2 and Bax together near the mitochondria. This assumption is in line with the observations that during apoptosis mitochondria accumulate around the nucleus in a microtubule and dynein-regulated manner^45^, which could be mediated via an interaction of nesprin-2G with the microtubular network via kinesin^46^ and dynein^40^. Furthermore, nesprin-2G interacts with the actin microfilament network^47^ and F-actin fibers assemble on the outer mitochondrial membrane^48^. F-actin fibers association with the mitochondria also increases in response to apoptotic stress^49^. In addition, association of nesprin-2 with mitochondria has been detected by immunogold electron microscopy^25^.

Apoptotic stress does not necessary culminate in cell death. Many tumor cells are resistant to various apoptotic stimuli. Our results show that in addition to Bax-induced MOMP, apoptotic stimuli can cause NPR/SIGRUNB and LINC complex alterations. It is therefore feasible that under conditions where the apoptotic process does not culminate in cell death, Bax-induced NPR/SIGRUNB and nesprin redistribution/degradation may affect cellular process regulated by the LINC complex, such as cell migration and gene expression^23,47,50,51^. Thus, our findings suggest that a new function of Bax may be the modulation of LINC complex-mediated cellular processes is stressed but living cells.

## Materials and methods

### Materials

All reagents were purchased from Sigma-Aldrich unless otherwise specified. Quinoline-VaL-Asp(OMe)-CH2-OPH (Q-VD-OPH) was purchased from Apex Biotechnology (Boston, MA, USA).

### Cell culture

Wild type (WT), Bax/Bak double knockout (DKO) and *caspase-9*^*−/−*^ 3T9 mouse embryonic fibroblasts (MEFs)^7^, Bax/Bak DKO HCT116 cells^37^ and Bax/Bak DKO U2OS cells^36^ were grown in high-glucose Dulbecco’s modified Eagle’s medium supplemented with 10% heat-inactivated fetal calf serum. Bax/Bak C16 DKO MEFs, stably expressing inducible Tet-on HA-Bax, were generated by transfecting pWHE655 HA-Bax into Bax/Bak DKO C3 MEFs, stably expressing the pWHE644 rtTA trans-activating vector^52^, followed by selection for 400 µg/ml Hygromycin B-resistant clones. For HA-Bax induction, Bax/Bak C16 MEFs were treated with 1 µg/ml doxycycline for 24 h.

### Plasmids

The expression vectors pEGFP (GFP), pEGFP-Bax (GFP-Bax), GFP-Bax P168A, FLAG-Bax, GFP-Bax L63E, GFP-Bax Δα5/6, GFP-Bax 63–65 A, GFP-Bax S184V were obtained as described^8^. His-Bax was prepared by PCR amplification of Bax from pEGFP-Bax as a template using the forward 5′ GTCAGAATTCATGGCTCACCACCACCACCACCACGACGGGTCCGGGGAGCAGCCC and reverse: 5′ CGTCTAGATCAGCCCATCTTCTTCCAGATGG primer set. The PCR product was cloned into EcoRI-XbaI sites of pcDNA3. pEGFP-Bax ΔN was prepared by inserting XhoI-EcoRI fragment containing Bax ΔN (generated by PCR using pEGFP-Bax as a template and the forward: 5′ GTCACTCGAGAAGACAGGGGCCCTTTTGCTTCAGG and reverse: 5′ GTCAGAATTCTTAGCCCATCTTCTTCCAGATGG primer set) into the XhoI-EcoRI sites of pEGFP; pWHE644 (rtTA- trans activating vector^53^) and pWHE655 (inducible vector^53^) were a gift from Christian Bernes (Friedrich-Loeffler-Institut, Jena, Germany). pWHE655 HA-Bax was prepared by PCR amplification, using the forward 5′ GTCAGAATTCTCCACCATGGCATACCC and reverse 5′ CGATGATATCTCAGCCCATCTTCTTCCAGATGG primers using pcDNA3 HA-Bax as a template. The PCR product was cloned into EcoRI- EcoRV sites of pWHE655. Generation of GFP-mini-nesprin-2G was described^39^. For the dimerization-dependent fluorescent protein exchange (ddFPX)^38^ experiments, RA-Bax (copy A; a monomer contains a chromophore that is quenched in the monomeric state) was generated by introducing Bax cDNA, obtained by PCR using eGFP-Bax as a template, into pAC-GFP-C1-RA using SacI and EcoRI restriction enzymes. GB-mini-nesprin-2G (copy B; a monomer that does not form a chromophore and acts to promote the fluorescence of copy A upon formation of the AB heterodimer) was prepared by PCR using the forward 5’-GCTCGAGGGATGGCCGCTAGCCCTGTG and the reverse 5’-GCGAATTCCTAGGTGGGAGGTGGCC primers using the GFP-mini-nesprin-2G plasmid as a template. The PCR product was cloned into the XhoI-EcoRI sites of pAcGFP-C1-GB. Mito-BFP^54^ was a gift from Dr. Gia K Voeltz, University of Colorado, USA.

### Transfection

Transfection was performed with TransIT-X2 (Mirus Bio LLC, Madison, WI USA) according to the manufacturer’s instructions. One day before transfection, cells were seeded at a density of 10^5^ cells per well in 12-well plates. When indicated, Q-VD-OPH (20 µM) was added 5 h after transfection.

### Immunofluorescence microscopy

10^5^ cells were grown on 18-mm cover slips coated with collagen. After various treatments, cells were fixed and stained with different antibodies and Hoechst 33258 dye, as described previously^55^. For mitochondrial staining, cells were incubated with 100 nM MitoTracker Red (Thermo Fisher Scientific, MA, USA) for 15 min at 37 °C before fixation, as described^9^. Fluorescent images were captured using a fluorescence microscope (EVOS Cell Imaging Systems, Thermo Fisher Scientific, MA, USA) or confocal microscope (LEICA TCS SP5 II) using Zeiss X63 NA 1.4 objective lens. To determine the number of cells exhibiting the redistribution effect, the fluorescently stained cells were counted under the fluorescence microscope.

### Antibodies

Antibodies used were mouse anti-Bax Clone 6A7 antibody (Ab) (# 556467) (BD Biosciences, CA, USA) at a dilution of 1:50 [for immunofluorescence (IF) staining and for Proximity Ligation Assay (PLA)]; rabbit anti-nesprin-2G Ab^32^ [1:100 (IF/PLA) or 1:1000 (for immunoblotting)]; rabbit anti-nesprin-1 Ab^31^ [1:100 (IF/PLA) or 1:1000 (immunoblotting)]; rabbit anti-nesprin-2 K2 Ab^31^ [1:100 (IF/PLA) or 1:1000 (immunoblotting)]; rabbit anti-nesprin-3 Ab^33^ [1:100 (IF), 1:1000 (immunoblotting)]; rabbit anti nesprin-2 MBS Ab (# MBS375177, MYBioSource.com, San Diego, CA, USA) [1:100 (IF/PLA)]; rabbit anti nesprin-2 Ab (# sc-365097) (Santa Cruz Biotechnology, Inc. Heidelberg, Germany) [1:200 (immunoblotting), 1:20 (immunoprecipitation (IP)]; mouse monoclonal anti-HA Ab (#H3663) (Sigma-Aldrich, MO, USA) [1:500 (immunoblotting)]; mouse monoclonal anti-Histidine TAG Ab (# MCA1396) (Bio-Rad Laboratories, Inc.CA, USA) [1:50 (IF/PLA)].

### Immunoblotting of LINC complex proteins

WT, Bax/Bak DKO and *caspase-9*^*−/−*^ MEFs were untreated or treated with 25 μM cisplatin in the presence or absence of 20 μM Q-VD-OPH. Twenty-four hours later, total cell lysates were harvested with hot 2× sample buffer (100 mM Tris-HCL, pH 6.8, 3.3% SDS, 16.6 mM DTT, 16.6% glycerol) supplemented with protease inhibitor cocktail (Calbiochem #539131) and heated at 95 °C for 5 min. One hundred microgram of proteins from each treatment was subjected to 4–15% Criterion TGX Precast Gradient Gel (Bio-Rad Laboratories, CA, USA) and electroblotted (3 h, 80 V) onto nitrocellulose membranes in the presence of blotting buffer (186 mM glycine, 25 mM Tris base, 0.08% SDS, 20% methanol). Each blot was blocked for 1 h in 10 mM Tris base, 150 mM NaCl containing 5% fat-free milk, then incubated for 16 h at 4 °C with the indicated primary antibody. Goat anti-mouse or anti-rabbit (1:10,000) IgG peroxidase conjugate (Jackson Immunoresearch Laboratories Inc.) were used as second antibody. The blots were developed using the Immobilon Crescendo Western HRP Substrate (Millipore, Merck, Massachusetts, USA). Normalization of the LINC complex proteins expression level was performed on Ponceau staining of blots derived from 10% SDS-PAGE loaded with the protein samples used for the analysis described in Fig. 4. The values obtained following immunoblotting were normalized separately to each of two different Ponceau‐stained bands (40 kDa and 80 kDa) obtained from the 10% SDS-PAGE and the renormalized results were presented as average of the two normalized values from 4 different independent experiments.

### IP and immunoblotting

For each co-IP experiment, 5 mg of the lysed cells [20 mM Tris (pH 7.5), 5 mM EDTA, 5 mM EGTA, 100 mM NaCl, and 1% CHAPS] supplemented with protease inhibitor cocktail were incubated at 4 °C for 1 h. After removal of cellular debris by centrifugation (10 min × 20,000×*g*, lysates were incubated with 50% anti-rabbit IgG-agarose beads (Sigma) together with 10 μg/ml rabbit anti-nesprin-2 Ab (Santa Cruz Biotechnology) for 18 h at 4 °C. The immunoprecipitated proteins or total extracts (100 μg protein) from each treatment were separated by 12.5% SDS–PAGE and electroblotted (1 h, 100 V) onto nitrocellulose membranes in the presence of blotting buffer (186 mM glycine, 25 mM Tris base, 20% methanol). Uniformity of sample loading was verified by Ponceau staining of the blots. Each blot was blocked for 30 min in 10 mM Tris base, 150 mM NaCl containing 5% fat-free milk, then incubated for 16 h at 4 °C with the primary Ab: mouse monoclonal anti-HA Ab or rabbit anti nesprin-2 Ab (Santa Cruz Biotechnology). Goat anti-rabbit or anti-mouse (1:10,000) IgG peroxidase conjugates were used as a second antibodies. The blots were developed using the Immobilon Crescendo Western HRP Substrate.

### Duolink PLA

The Duolink PLA assay^56^ was performed using Duolink in situ PLUS and MINUS probes and Duolink in situ detection reagents FarRed according to manufacturer’s instructions (Sigma-Aldrich). At the end of the procedure the slides were mounted with a coverslip using Duolink^®^ In Situ Mounting Medium which contains 4,6-Diamidino-2-phenylindole, dihydrochloride (DAPI) for nuclear staining. Imaging was performed by fluorescence or confocal microscopy as above. Duolink signal appears as dots.

### ddFPX

U2OS Bak/Bak DKO cells were seeded in 8-well chambers (Ibidi) and transfected with the RA-Bax, GB-mini-nesprin-2G and mito-BFP. Q-VD-OPH (20 µM) was added to the cells 5 h before transfection. Cells were imaged 24 h after transfection in the red and the blue channels. Images were acquired with a ×63 water objective in an LSM780 confocal microscope equipped with an incubator at 37 °C and 5% CO_2_. We quantified manually the number of cells in which positive ddFPX was observed using Image J. Since we cannot detect the cells that were transfected with RA- and GB- plasmids unless ddFPX is positive, we normalized the interacting cells by the number of cells exhibiting mito-BFP fluorescence, assuming that integration of the three plasmids occurred simultaneously in each cell. Each experiment was repeated three times and a minimum of 50 mito-BFP positive cells were quantified in each condition.

### Statistical analysis

Statistical significance was determined using Student’s *t*-test or by one or two-way ANOVA. Values of *p* < 0.05 were considered statistically significant. Data were expressed as mean values ± S.E.M. Sample size was chosen according to well-established rules in the literature, as well as our previous experience.

## Supplementary information

Supplemental Figure Legends

Supplemental Figure 1

Supplemental Figure 2

Supplemental Figure 3

Supplemental Figure 4

Supplemental Figure 5

Supplemental Figure 6

Supplemental Figure 7
