# Supplementary material for: Apoptotic stress induces Bax-dependent, caspase-independent redistribution of LINC complex nesprins
Source: Cell Death Discov. 2020 Sep 18;6:90. doi: 10.1038/s41420-020-00327-6 (PMC7501853; doi:10.1038/s41420-020-00327-6)
Supplement: Supplementary file 1 — Supplemental Figure Legends [file 41420_2020_327_MOESM1_ESM.docx]

**Apoptotic stress induces Bax-dependent, caspase-independent redistribution of LINC complex nesprins**

Liora Lindenboim^1^, Dan Grozki^1^, Ayelet R Amsalem-Zafran^1^, Aida Peña-Blanco^2^, Gregg G Gundersen^3^, Christoph Borner^4,5^, Didier Hodzic^6^, Ana J Garcia- Sáez^7^, Howard J Worman^3,8^ and Reuven Stein^1^*

**Supplemental Figure Legends**

**Figure S1**. **Nesprin-1 and nesprin-2 redistribution in *caspase-9^−/−^* MEFs.** *Caspase-9^−/−^* MEFs were untreated (Con) or treated for 24 h with 25 µM cisplatin (Cis) or 17 h with 100 nM staurosporine (STS). Then the cells were stained for nesprin proteins as described in Figure 1. (a) Representative photomicrographs of the staining of the different nesprin proteins is shown in a. Bar = 50 µm. (b) Quantification of the redistribution of each nesprin protein expressed as described in Figure 1. The values are presented as mean ± SEM (error bars) (n = 3) (**p* < 0.05, two tailed student’s *t* test).

**Figure S2. Re-expression of Bax in DKO MEFs promotes cisplatin-induced caspase-independent nesprin-1 and nesprin-2 redistribution.**

Bax/Bak C16 DKO MEFs (C16 DKO) were untreated (Con) or treated for 24 h with doxycycline to induce Bax expression (Dox), or treated for 24 h with doxycycline followed by treatment with cisplatin in the presence of Q-VD-OPH for additional 24 h (Dox+Cis+QVD). Cells were co-stained for the nesprin proteins (as described in Figure 1) and for Bax (anti-HA ab). Number of cells exhibiting redistribution of nesprin-1, nesprin-2 and nesprin-3 from Bax-expressing cells was determined microscopically. Representative photomicrographs are shown for nesprin-2G (a), nesprin-2 K2 (b), nepsrin-1 (c), nesprin-3 (d). Bar = 25 µm. (e) Quantification of the redistribution of each nesprin protein is shown as described in Figure 1. Values are presented as mean ± SEM (error bars) (n = 3) (**p* < 0.05, ****p* < 0.002, two tailed student’s *t* test).

**Figure S3. Images of Ponceau staining of blots of the different protein samples used for normalizing LINC protein expression**. Protein samples used for the analysis described in Figure 3, from four independent experiments, were loaded on 10% SDS-PAGE gels, blotted and stained with Ponceau. The images show the 40 kDa and 80 kDa Ponceau bands that their normalized average values were used for normalization of the results presented in Figure 3.

**Figure S4. Stress-induced redistributed nesprin-2 co-localizes with active Bax and mitochondria.** *Caspase-9^−/−^* MEFs were treated with cisplatin for 24 h, stained with MitoTracker Red (mitochondrial marker), fixed and stained with anti-Bax (6A7) (activated Bax) and pan anti-nesprin-2 (MBS375177) Ab. The localization of the redistributed nesprin-2 in relation to the mitochondria and activated Bax was determined by confocal microscopy. Photomicrographs were captured from the same field to visualized separately active Bax (green), nesprin-2 (blue) and mitochondria (red) fluorescence. The images shown are from a representative cell out of 25 cells captured in two independent experiments. Bar = 25 and 50 µm for lower and upper panels respectively.

**Figure S5.** **Bax/Bak DKO U2OS and HCT116 cells express endogenous nesprin-2G.** (a) Bax/Bak DKO U2OS and HCT116 cells were stained for nesprin-2G and visualized by fluorescence microscopy. The images of each cell line (upper and lower panels) represent the same field visualized separately for detecting nesprin-2G and nuclei staining (Bar = 25 µm). (b) Immunoblots of lysates of Bax/Bak DKO U2OS and HCT116 cells using anti nesprin-2G Ab.

**Figure S6. Bax/nesprin-2G interaction in** **Bax/Bak DKO U2OS and HCT116 cells; Duolink-PLA approach**. Bax/Bak DKO U2OS and HCT116 cells were transiently transfected with GFP or GFP-Bax expression vectors and the interaction between Bax and nesprin-2G was examined by Duolink-PLA, using anti-Bax (6A7) and anti-nesprin-2G Ab. (a, b) The photomicrographs shown for DKO U2OS (a) DKO HCT116 (b) are from the same field visualized separately for GFP fluorescence (upper panel), Duolink signal (middle panel) and DAPI-stained nuclei (lower panel). The results presented are from a representative experiment (n = 4). Bar = 50 µm (c, d) Quantification of the Duolink signal in DKO U2OS (c) DKO HCT116 (d) cells. The results are expressed as the number of dots normalized to cell size in transfected cell (at least 20 cells of each treatment) and are presented as mean ± SEM (error bars) (n = 4). (***p* < 0.02, two tailed student’s *t* test).

**Figure S7. Interaction between GFP-mini-nesprin-2G and FLAG-Bax; Duolink-PLA approach.** (a) Visualization of GFP-mini-nesprin-2G (GFP-mini-nesp-2G) expression pattern in transfected Bax/Bak DKO MEFs. Bax/Bak DKO MEFs were transfected with GFP-mini-nesprin-2G expression vector. 24 h later the cells were fixed, stained with Hoechst dye and visualized by fluorescence microscopy. The results shown are from a representative cell (from 3 independent experiments). The photomicrographs shown are from the same field visualized separately for detecting GFP-mini-nesprin-2G and nuclei staining. Bar = 25 µm (b) Interaction between GFP-mini-nesprin-2G and FLAG-Bax. Bax/Bak DKO MEFs were transiently co-transfected FLAG-Bax with either GFP or GFP-mini-nesprin-2G expression vectors in the presence of Q-VD-OPH. Interaction was assessed by Duolink-PLA using the pan anti-nesprin-2 K2 and anti-Bax (6A7) Ab. The results are expressed as the number of dots normalized to cell size in transfected cell (at least 20 cells in each treatment) and are presented as mean ± SEM (error bars) (n = 3) (****p* < 0.002, two tailed student’s *t* test).
