# Supplementary figures and images for: Apoptotic stress induces Bax-dependent, caspase-independent redistribution of LINC complex nesprins
Source: Cell Death Discov. 2020 Sep 18;6:90. doi: 10.1038/s41420-020-00327-6 (PMC7501853; doi:10.1038/s41420-020-00327-6)

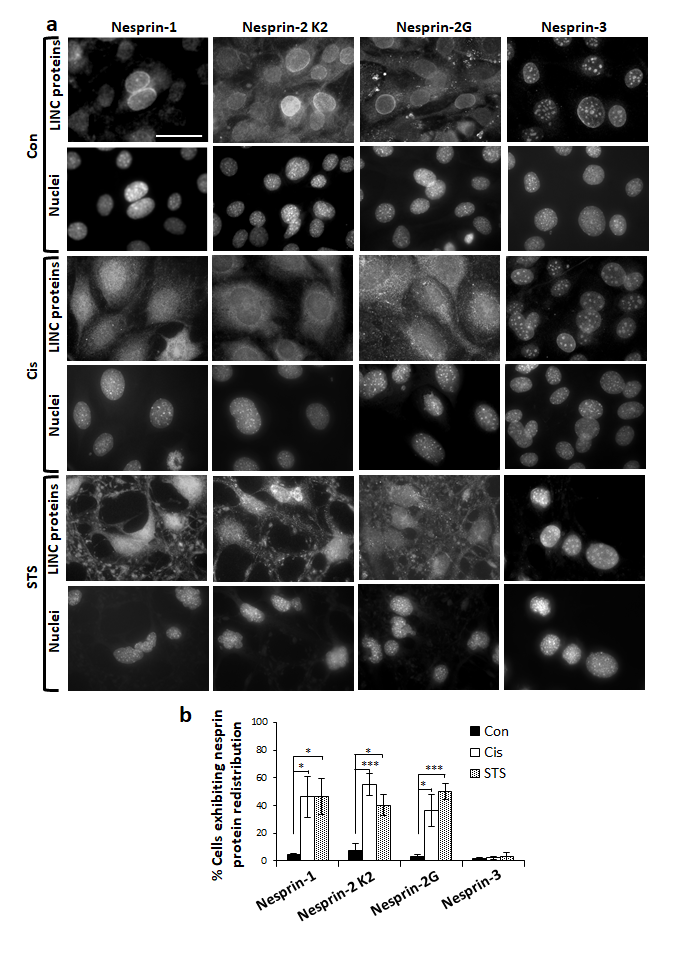

Supplement: Supplementary file 2 — Supplemental Figure 1 [file 41420_2020_327_MOESM2_ESM.tif]

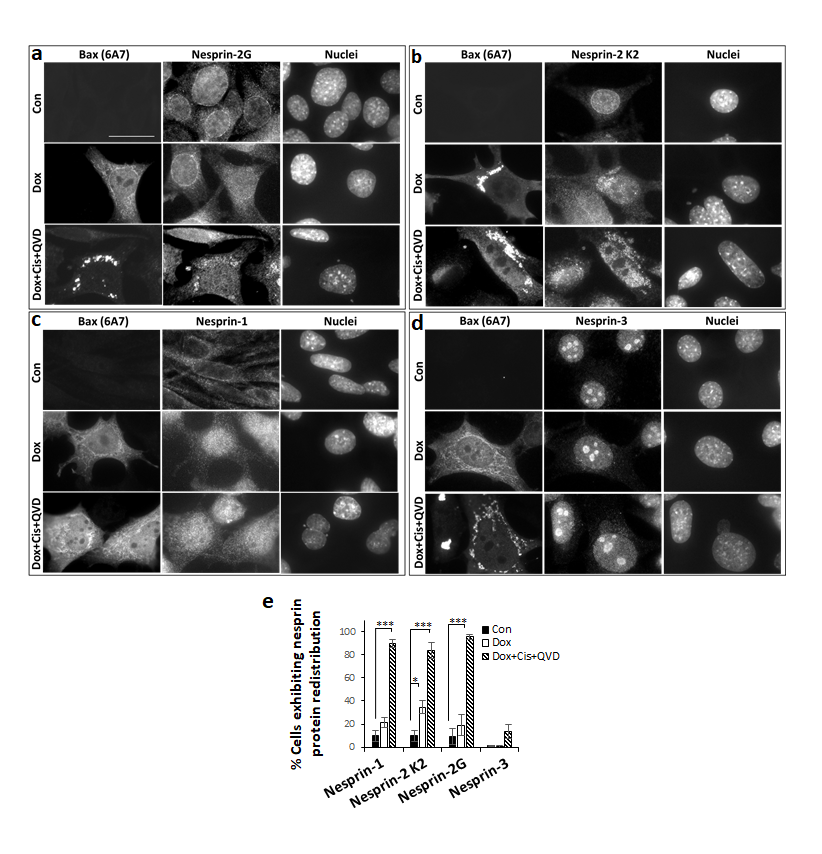

Supplement: Supplementary file 3 — Supplemental Figure 2 [file 41420_2020_327_MOESM3_ESM.tif]

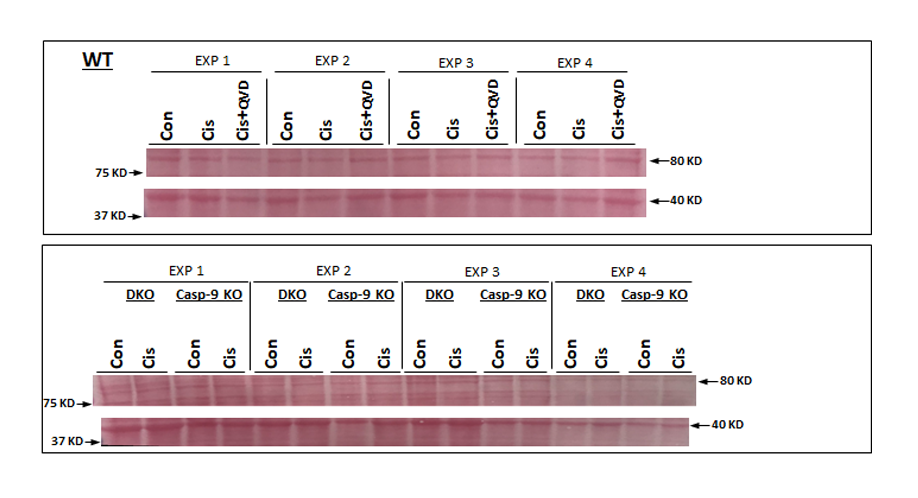

Supplement: Supplementary file 4 — Supplemental Figure 3 [file 41420_2020_327_MOESM4_ESM.tif]

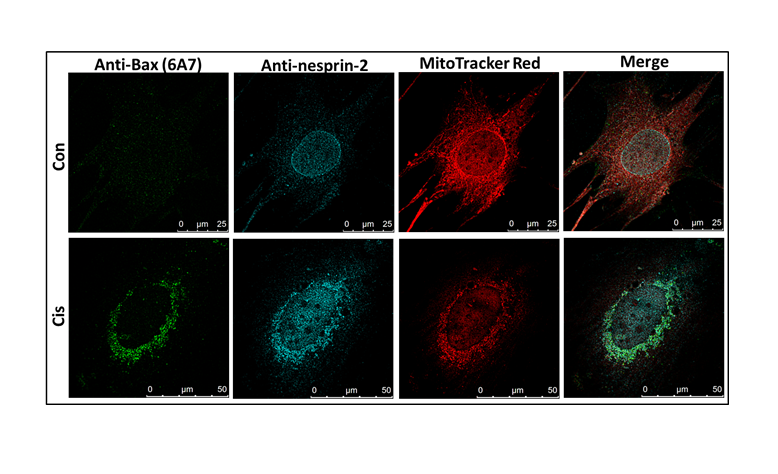

Supplement: Supplementary file 5 — Supplemental Figure 4 [file 41420_2020_327_MOESM5_ESM.tif]

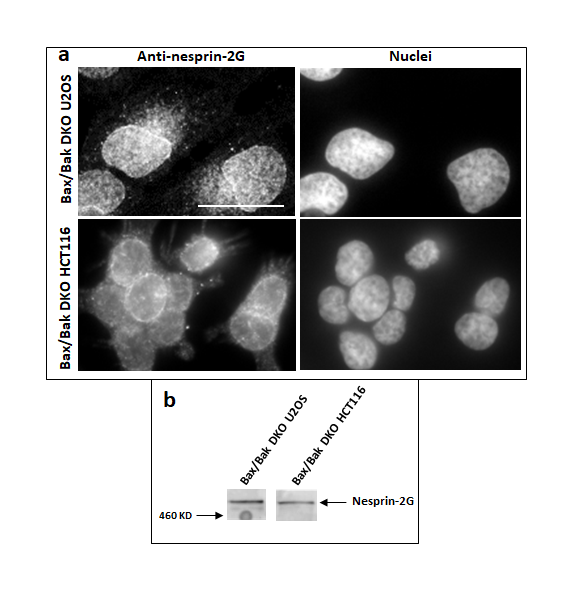

Supplement: Supplementary file 6 — Supplemental Figure 5 [file 41420_2020_327_MOESM6_ESM.tif]

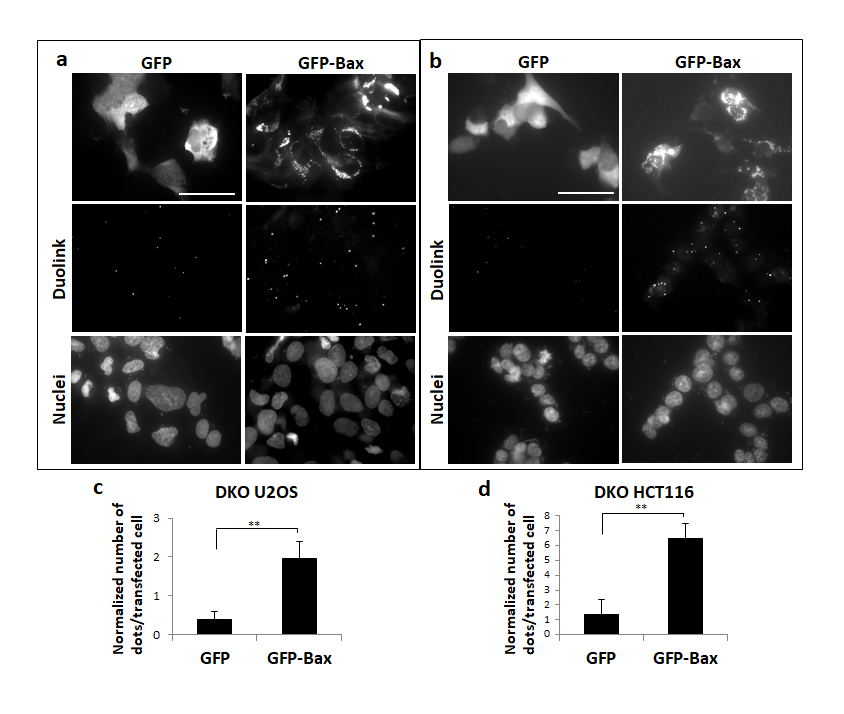

Supplement: Supplementary file 7 — Supplemental Figure 6 [file 41420_2020_327_MOESM7_ESM.tif]

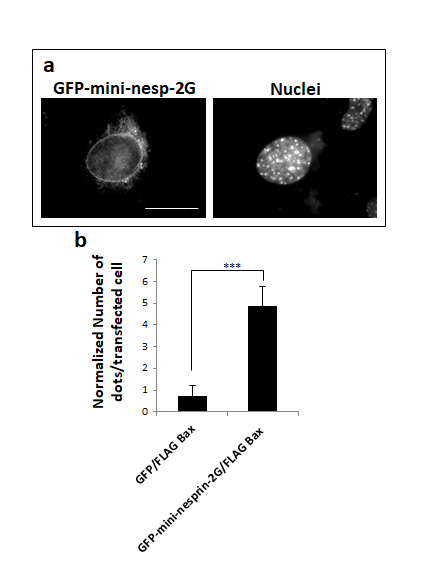

Supplement: Supplementary file 8 — Supplemental Figure 7 [file 41420_2020_327_MOESM8_ESM.tif]
